# Supplementary material for: Six-year retrospective analysis of the epidemiology and risk factors of multidrug-resistant bloodstream infections in oncology patients in Jiangxi, China
Source: Microbiol Spectr. 2025 Aug 14;13(10):e01468-25. doi: 10.1128/spectrum.01468-25 (PMC12502737; doi:10.1128/spectrum.01468-25)
Supplement: Supplemental material — Fig. S1; Tables S1 to S8. [file spectrum.01468-25-s0001.pdf]

a

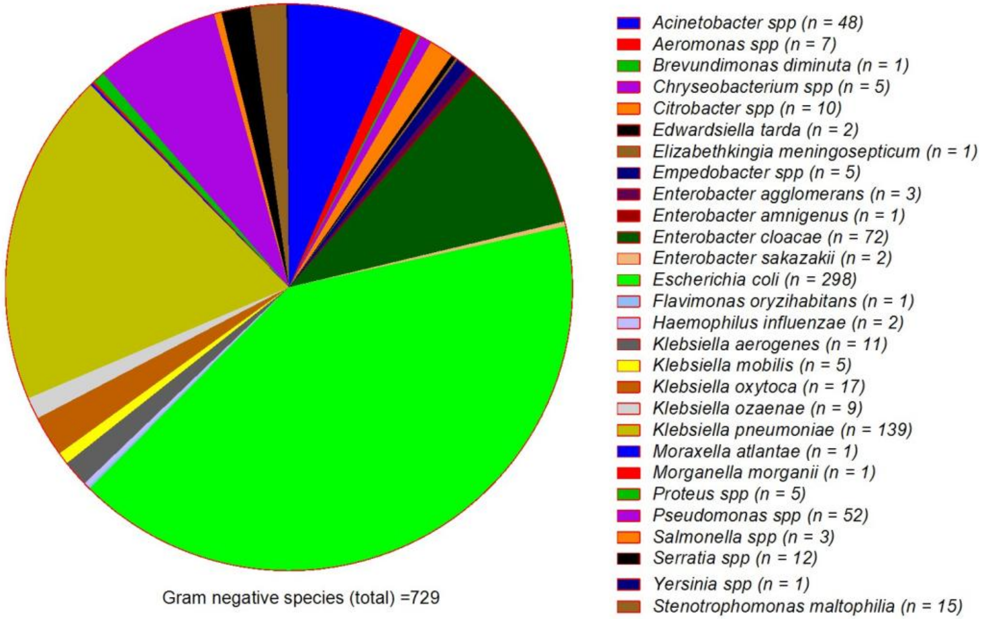


Gram negative species (total) =729

b

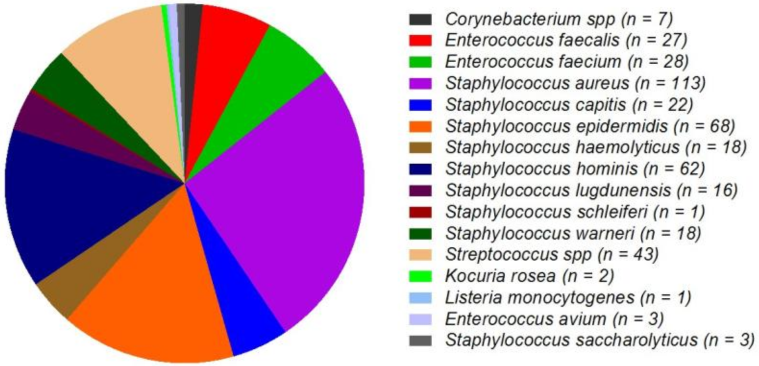


Gram positive species (total=432)

Figure S1: Bacterial count: a) The number and percentages of gram-negative bacteria causing Bloodstream Infections (BSI) in cancer patients. b) The number and percentages of gram-positive bacteria causing Bloodstream Infections (BSI) in cancer patients.

Table S1: Year wise distribution of BSIs episodes, and number of bacteria in oncological patients.

| Years | Patients | Episode | Monomicrobial | Polymicrobial (2 bacetria per episode) | Polymicrobial (3 bacteria per episode) |
|---|---|---|---|---|---|
| **2019** | 141 | 149 | 140 | 9 | 0 |
| **2020** | 161 | 196 | 185 | 11 | 0 |
| **2021** | 145 | 183 | 174 | 9 | 0 |
| **2022** | 157 | 162 | 155 | 7 | 0 |
| **2023** | 179 | 226 | 208 | 17 | 1 |
| **2024** | 171 | 179 | 170 | 7 | 2 |
| **Total** | **954** | **1095** | **1032** | **60** | **3** |

Table S2: List of bacterial species identified in polymicrobial bloodstream infection (BSI) episodes.

| Number of episodes | Bacteria |
|---|---|
| 1 | *[Enterobacter cloacae, Enterococcus faecium]* |
| 1 | *[Serratia marcescens, Serratia proteamaculans]* |
| 3 | *[Enterococcus faecium, Escherichia coli]* |
| 1 | *[Klebsiella mobilis, Klebsiella ozaenae]* |
| 1 | *[Enterobacter amnigenus, Escherichia coli]* |
| 1 | *[Escherichia coli, Streptococcus, beta-haem. Group C]* |
| 1 | *[Acinetobacter baumannii, Streptococcus constellatus]* |
| 1 | *[Staphylococcus lugdunensis, Staphylococcus warneri]* |
| 1 | *[Citrobacter braakii, Streptococcus mutans]* |
| 2 | *[Enterococcus faecium, Klebsiella pneumoniae]* |
| 1 | *[Enterobacter cloacae, Staphylococcus aureus]* |
| 1 | *[Staphylococcus epidermidis, Staphylococcus hominis ss. hominis]* |
| 1 | *[Enterococcus faecalis, Pseudomonas aeruginosa]* |
| 1 | *[Staphylococcus aureus, Staphylococcus hominis ss. hominis]* |
| 1 | *[Acinetobacter baumannii, Klebsiella pneumoniae]* |
| 1 | *[Corynebacterium jeikeium, Staphylococcus hominis ss. hominis]* |
| 1 | *[Acinetobacter baumannii, Flavimonas oryzihabitans]* |
| 1 | *[Enterococcus faecalis, Escherichia coli]* |
| 1 | *[Acinetobacter lwoffii, Staphylococcus epidermidis]* |
| 1 | *[Pseudomonas maltophilia, Staphylococcus hominis ss. hominis]* |

| | |
|---|---|
| 1 | *[Citrobacter freundii, Streptococcus viridans, alpha-hem.]* |
| 3 | *[Escherichia coli, Staphylococcus aureus]* |
| 2 | *[Citrobacter freundii, Escherichia coli]* |
| 1 | *[Aeromonas caviae, Escherichia coli]* |
| 1 | *[Klebsiella ozaenae, Staphylococcus warneri]* |
| 1 | *[Empedobacter brevis, Klebsiella pneumoniae]* |
| 1 | *[Enterococcus faecium, Pseudomonas aeruginosa]* |
| 1 | *[Pseudomonas aeruginosa, Streptococcus pneumoniae]* |
| 1 | *[Staphylococcus hominis ss. hominis, Staphylococcus lugdunensis]* |
| 1 | *[Enterobacter cloacae, Escherichia coli]* |
| 1 | *[Acinetobacter baumannii, Pseudomonas fluorescens]* |
| 1 | *[Escherichia coli, Klebsiella mobilis]* |
| 3 | *[Enterococcus faecalis, Klebsiella pneumoniae]* |
| 1 | *[Escherichia coli, Pseudomonas putrefaciens]* |
| 1 | *[Enterococcus faecalis, Staphylococcus capitis]* |
| 1 | *[Klebsiella pneumoniae, Streptococcus dysgalactiae]* |
| 1 | *[Escherichia coli, Streptococcus pneumoniae]* |
| 1 | *[Acinetobacter baumannii, Enterococcus faecium, Pseudomonas maltophilia]* |
| 2 | *[Staphylococcus aureus, Staphylococcus epidermidis]* |
| 1 | *[Acinetobacter baumannii, Klebsiella oxytoca, Enterobacter cloacae]* |
| 3 | *[Escherichia coli, Klebsiella pneumoniae]* |
| 2 | *[Escherichia coli, Stenotrophomonas maltophilia]* |

| | |
|---|---|
| 2 | *[Escherichia coli, Klebsiella oxytoca]* |
| 1 | *[Klebsiella pneumoniae, Enterococcus faecium, Staphylococcus epidermidis]* |
| 1 | *[Klebsiella aerogenes, Klebsiella pneumoniae]* |
| 1 | *[Chryseobacterium indologenes, Klebsiella pneumoniae]* |
| 1 | *[Klebsiella aerogenes, Staphylococcus epidermidis]* |
| 1 | *[Enterobacter cloacae, Pseudomonas aeruginosa]* |

Table S3: Demographical and clinical characteristics of gram-negative and gram-positive bacteria in the current study

| Variable | Total (n) | Total (%) | G -ve (n) | G -ve (%) | G +ve (n) | G +ve (%) | chi square | p value |
|---|---|---|---|---|---|---|---|---|
| **Gender** | | | | | | | | |
| Male | 676 | 58.23 | 410 | 60.65 | 266 | 39.35 | 3.17 | 0.07 |
| Female | 485 | 41.77 | 319 | 65.77 | 166 | 34.23 | 3.17 | 0.07 |
| **Age (Median (IQR)** | 59 | (50 to 67) | 59 | (50 to 67) | 58 | (49 to 67) | -1# | 0.17* |
| **Occupation** | | | | | | | | |
| Jobless/Unemployed/Retired | 349 | 30.06 | 212 | 60.74 | 137 | 39.26 | 0.89 | 0.34 |
| Laborer/Manual Worker | 650 | 55.99 | 420 | 64.62 | 230 | 35.38 | 2.11 | 0.15 |
| Freelancer/Self-Employed | 94 | 8.1 | 59 | 62.77 | 35 | 37.23 | 2.680e-005, | 1 |
| Professional and Technical | 68 | 5.86 | 38 | 55.88 | 30 | 44.12 | 1.48 | 0.22 |
| **Education background** | | | | | | | | |
| No Formal Education | 317 | 27.3 | 192 | 60.57 | 125 | 39.43 | 0.92 | 0.34 |
| Basic Education | 386 | 33.25 | 258 | 66.84 | 128 | 33.16 | 4.06 | **0.04** |
| Intermediate Education | 196 | 16.88 | 128 | 65.31 | 68 | 34.69 | 0.64 | 0.42 |
| Secondary Education | 194 | 16.71 | 110 | 56.7 | 84 | 43.3 | 3.7 | 0.05 |
| Higher Education | 68 | 5.86 | 41 | 60.29 | 27 | 39.71 | 0.19 | 0.66 |
| **Type of infection** | | | | | | | | |
| Hospital acquired | 950 | 81.83 | 573 | 60.32 | 377 | 39.68 | 13.7 | **<0.001** |
| Community acquired | 211 | 18.17 | 156 | 73.93 | 55 | 26.07 | 13.7 | **<0.001** |
| **Cancer treatment** | | | | | | | | |
| Chemotherapy | 669 | 57.62 | 416 | 62.18 | 253 | 37.82 | 0.25 | 0.62 |
| Radiotherapy | 127 | 10.94 | 79 | 62.2 | 48 | 37.8 | 0.02 | 0.88 |

| | | | | | | | | |
|---|---|---|---|---|---|---|---|---|
| Targeted therapy | 230 | 19.81 | 142 | 61.74 | 88 | 38.26 | 0.14 | 0.71 |
| Immunotherapy | 102 | 8.79 | 69 | 67.65 | 33 | 32.35 | 1.13 | 0.29 |
| **Procedures and Interventions** | | | | | | | | |
| CVC | 158 | 13.61 | 88 | 55.7 | 70 | 44.3 | 3.94 | 0.05 |
| Urinary catheter | 263 | 22.65 | 162 | 61.6 | 101 | 38.4 | 0.21 | 0.65 |
| PICC | 196 | 16.88 | 121 | 61.73 | 75 | 38.27 | 0.11 | 0.74 |
| Safety IV catheter | 669 | 57.62 | 410 | 61.29 | 259 | 38.71 | 1.53 | 0.22 |
| Ventilator | 48 | 4.13 | 22 | 45.83 | 26 | 54.17 | 6.16 | **0.01** |
| Blood transfusion | 274 | 23.6 | 178 | 64.96 | 96 | 35.04 | 0.72 | 0.39 |
| Biopsies | 118 | 10.16 | 86 | 72.88 | 32 | 27.12 | 5.72 | **0.02** |
| Intra-abdominal catheter | 178 | 15.33 | 133 | 74.72 | 45 | 25.28 | 12.8 | **<0.001** |
| Parenteral nutrition | 339 | 29.2 | 220 | 64.9 | 119 | 35.1 | 0.91 | 0.34 |
| Nasogastric feeding | 74 | 6.37 | 35 | 47.3 | 39 | 52.7 | 8.12 | **<0.001** |
| Surgery | 137 | 11.8 | 57 | 41.61 | 80 | 58.39 | 29.84 | **<0.001** |
| **Infection and infectious diseases** | | | | | | | | |
| Sepsis | 570 | 49.1 | 361 | 63.33 | 209 | 36.67 | 0.14 | 0.71 |
| Septic shock | 82 | 7.06 | 66 | 80.49 | 16 | 19.51 | 11.83 | **<0.001** |
| Hepatitis B | 154 | 13.26 | 98 | 63.64 | 56 | 36.36 | 0.05 | 0.82 |
| Pneumonia | 185 | 15.93 | 109 | 58.92 | 76 | 41.08 | 1.41 | 0.23 |
| Urinary tract infection | 73 | 6.29 | 57 | 78.08 | 16 | 21.92 | 7.8 | **0.01** |
| Biliary tract infection | 93 | 8.01 | 74 | 79.57 | 19 | 20.43 | 12.18 | **<0.001** |
| **Conditions and complications** | | | | | | | | |
| Anemia | 325 | 27.99 | 210 | 64.62 | 115 | 35.38 | 0.64 | 0.42 |
| Hypertension | 177 | 15.25 | 110 | 62.15 | 67 | 37.85 | 0.04 | 0.85 |

| | | | | | | | | |
|---|---|---|---|---|---|---|---|---|
| Diabetes | 104 | 8.96 | 63 | 60.58 | 41 | 39.42 | 0.24 | 0.62 |
| Thrombosis | 40 | 3.45 | 15 | 37.5 | 25 | 62.5 | 11.34 | **<0.001** |
| Myelosuppression | 306 | 26.36 | 185 | 60.46 | 121 | 39.54 | 0.97 | 0.33 |
| Hypoproteinemia | 429 | 36.95 | 292 | 68.07 | 137 | 31.93 | 8.1 | **<0.001** |
| Nausea and Vomiting | 38 | 3.27 | 27 | 71.05 | 11 | 28.95 | 1.15 | 0.28 |
| Radiation dermatitis | 19 | 1.64 | 14 | 73.68 | 5 | 26.32 | 0.98 | 0.32 |
| Respiratory failure | 48 | 4.13 | 27 | 56.25 | 21 | 43.75 | 0.92 | 0.34 |
| Ascites | 121 | 10.42 | 79 | 65.29 | 42 | 34.71 | 0.36 | 0.55 |
| Leukopenia | 125 | 10.77 | 76 | 60.8 | 49 | 39.2 | 0.24 | 0.63 |
| Cerebral infarction | 38 | 3.27 | 24 | 63.16 | 14 | 36.84 | 0 | 0.96 |
| Electrolyte disorders | 222 | 19.12 | 142 | 63.96 | 80 | 36.04 | 0.16 | 0.69 |
| Neutropenia | 157 | 13.52 | 106 | 67.52 | 51 | 32.48 | 1.74 | 0.19 |
| Neutrophilia | 475 | 40.91 | 316 | 66.53 | 159 | 33.47 | 4.8 | **0.03** |
| **Hospital stays (in days)** | 20 | (13 to 31) | 20 | (12 to 31) | 20 | (13 to 31) | 1# | 0.3* |
| **Mortality (30 days)** | 58 | 5 | 35 | 60.34 | 23 | 39.66 | 0.16 | 0.69 |

*Mann-Whitney U, #Difference: Hodges-Lehmann

Table S4: Antibiotic susceptibility profiles of *Escherichia coli*, *Klebsiella* Species, and *Enterobacter* Species in the current study.

| **Antibiotics** | ***Escherichia coli*** | | | | ***Klebsiella* Species** | | | | ***Enterobacter* Species** | | | |
|---|---|---|---|---|---|---|---|---|---|---|---|---|
| | T | R (%) | I (%) | S (%) | T | R (%) | I (%) | S (%) | T | R (%) | I (%) | S (%) |
| Ampicillin | 204 | 180 (88.24) | 3 (1.47) | 21 (10.29) | 132 | 117 (88.64) | 11 (8.33) | 4 (3.03) | 57 | 52 (91.23) | 3 (5.26) | 2 (3.51) |
| Ticarcillin/Clavulanic acid | 212 | 30 (14.15) | 70 (33.02) | 112 (52.83) | 130 | 28 (21.54) | 15 (11.54) | 87 (66.92) | 57 | 3 (5.26) | 3 (5.26) | 51 (89.47) |
| Piperacillin/Tazobactam | 169 | 15 (8.88) | 8 (4.73) | 146 (86.39) | 130 | 22 (16.92) | 7 (5.38) | 101 (77.69) | 57 | 3 (5.26) | 1 (1.75) | 53 (92.98) |
| Piperacillin | 201 | 136 (67.66) | 0 (0) | 65 (32.34) | 130 | 41 (31.54) | 0 (0) | 89 (68.46) | 57 | 11 (19.3) | 0 (0) | 46 (80.7) |
| Ampicillin/Sulbactam | 203 | 67 (33) | 68 (33.5) | 68 (33.5) | 132 | 41 (31.06) | 24 (18.18) | 67 (50.76) | 56 | 21 (37.5) | 23 (41.07) | 12 (21.43) |
| Cefuroxime | 222 | 124 (55.86) | 26 (11.71) | 72 (32.43) | 135 | 44 (32.59) | 7 (5.19) | 84 (62.22) | 54 | 17 (31.48) | 8 (14.81) | 29 (53.7) |
| Ceftriaxone | 296 | 161 (54.39) | 1 (0.34) | 134 (45.27) | 177 | 51 (28.81) | 0 (0) | 126 (71.19) | 79 | 12 (15.19) | 0 (0) | 67 (84.81) |
| Ceftazidime | 296 | 62 (20.95) | 24 (8.11) | 210 (70.95) | 177 | 33 (18.64) | 10 (5.65) | 134 (75.71) | 78 | 7 (8.97) | 3 (3.85) | 68 (87.18) |
| Cefepime | 296 | 45 (15.2) | 33 (11.15) | 218 (73.65) | 177 | 21 (11.86) | 9 (5.08) | 147 (83.05) | 79 | 3 (3.8) | 1 (1.27) | 75 (94.94) |
| Cefazolin | 296 | 172 (58.11) | 0 (0) | 124 (41.89) | 177 | 75 (42.37) | 0 (0) | 102 (57.63) | 80 | 73 (91.25) | 0 (0) | 7 (8.75) |
| Doripenem | 288 | 7 (2.43) | 1 (0.35) | 280 (97.22) | 174 | 12 (6.9) | 4 (2.3) | 158 (90.8) | 79 | 3 (3.8) | 0 (0) | 76 (96.2) |
| Ertapenem | 288 | 7 (2.43) | 0 (0) | 281 (97.57) | 175 | 16 (9.14) | 0 (0) | 159 (90.86) | 79 | 3 (3.8) | 1 (1.27) | 75 (94.94) |
| Imipenem | 297 | 8 (2.69) | 1 (0.34) | 288 (96.97) | 178 | 14 (7.87) | 2 (1.12) | 162 (91.01) | 80 | 2 (2.5) | 1 (1.25) | 77 (96.25) |
| Meropenem | 295 | 8 (2.71) | 0 (0) | 287 (97.29) | 177 | 14 (7.91) | 2 (1.13) | 161 (90.96) | 78 | 2 (2.56) | 1 (1.28) | 75 (96.15) |
| Aztreonam | 241 | 65 (26.97) | 19 (7.88) | 157 (65.15) | 155 | 30 (19.35) | 5 (3.23) | 120 (77.42) | 67 | 10 (14.93) | 1 (1.49) | 56 (83.58) |
| Tobramycin | 296 | 69 (23.31) | 36 (12.16) | 191 (64.53) | 177 | 17 (9.6) | 5 (2.82) | 155 (87.57) | 79 | 3 (3.8) | 2 (2.53) | 74 (93.67) |
| Amikacin | 296 | 9 (3.04) | 2 (0.68) | 285 (96.28) | 177 | 6 (3.39) | 0 (0) | 171 (96.61) | 79 | 1 (1.27) | 0 (0) | 78 (98.73) |
| Gentamicin | 296 | 96 (32.43) | 8 (2.7) | 192 (64.86) | 177 | 24 (13.56) | 1 (0.56) | 152 (85.88) | 78 | 4 (5.13) | 0 (0) | 74 (94.87) |
| Tigecycline | 296 | 0 | 0 | 296 | 177 | 1 | 0 | 176 | 80 | 0 | 0 | 80 |

| | | | | | | | | | | | | |
|---|---|---|---|---|---|---|---|---|---|---|---|---|
| | | (0) | (0) | (100) | | (0.56) | (0) | (99.44) | | (0) | (0) | (100) |
| Minocycline | 296 | 37 (12.5) | 24 (8.11) | 235 (79.39) | 176 | 19 (10.8) | 4 (2.27) | 153 (86.93) | 78 | 9 (11.54) | 1 (1.28) | 68 (87.18) |
| Tetracycline | 296 | 186 (62.84) | 2 (0.68) | 108 (36.49) | 177 | 50 (28.25) | 4 (2.26) | 123 (69.49) | 80 | 15 (18.75) | 5 (6.25) | 60 (75) |
| Nitrofurantoin | 296 | 1 (0.34) | 5 (1.69) | 290 (97.97) | 177 | 3 (1.69) | 20 (11.3) | 154 (87.01) | 79 | 6 (7.59) | 27 (34.18) | 46 (58.23) |
| Ciprofloxacin | 297 | 169 (56.9) | 0 (0) | 128 (43.1) | 179 | 32 (17.88) | 0 (0) | 147 (82.12) | 80 | 10 (12.5) | 0 (0) | 70 (87.5) |
| Levofloxacin | 296 | 158 (53.38) | 0 (0) | 138 (46.62) | 177 | 22 (12.43) | 0 (0) | 155 (87.57) | 79 | 12 (15.19) | 0 (0) | 67 (84.81) |
| Trimethoprim/Sulfamethoxazole | 296 | 161 (54.39) | 0 (0) | 135 (45.61) | 177 | 52 (29.38) | 0 (0) | 125 (70.62) | 80 | 14 (17.5) | 0 (0) | 66 (82.5) |

T = number of tested isolates, R = resistant, I = intermediate, S = susceptible.

Table S5: Antibiotic susceptibility profiles of *Acinetobacter* species, *Pseudomonas* species and Other gram-negative species in the current study.

| **Antibiotics** | ***Acinetobacter* Species** | | | | ***Pseudomonas* Species** | | | | **Other gram-negative species** | | | |
|---|---|---|---|---|---|---|---|---|---|---|---|---|
| | T | R (%) | I (%) | S (%) | T | R (%) | I (%) | S (%) | T | R (%) | I (%) | S (%) |
| Ampicillin | | | | | | | | | 22 | 18 (81.82) | 0 (0) | 4 (18.18) |
| Ticarcillin/Clavulanic acid | 27 | 1 (3.7) | 1 (3.7) | 25 (92.59) | 36 | 8 (22.22) | 13 (36.11) | 15 (41.67) | 43 | 9 (20.93) | 9 (20.93) | 25 (58.14) |
| Piperacillin/Tazobactam | 34 | 1 (2.94) | 1 (2.94) | 32 (94.12) | 32 | 3 (9.38) | 2 (6.25) | 27 (84.38) | 33 | 4 (12.12) | 1 (3.03) | 28 (84.85) |
| Piperacillin | 34 | 2 (5.88) | 2 (5.88) | 30 (88.24) | 36 | 3 (8.33) | 2 (5.56) | 31 (86.11) | 34 | 11 (32.35) | 1 (2.94) | 22 (64.71) |
| Ampicillin/Sulbactam | 34 | 2 (5.88) | 0 (0) | 32 (94.12) | | | | | 20 | 9 (45) | 5 (25) | 6 (30) |
| Cefuroxime | | | | | | | | | 22 | 12 (54.55) | 1 (4.55) | 9 (40.91) |
| Ceftriaxone | 48 | 6 (12.5) | 9 (18.75) | 33 (68.75) | | | | | 51 | 9 (17.65) | 0 (0) | 42 (82.35) |
| Ceftazidime | 48 | 5 (10.42) | 1 (2.08) | 42 (87.5) | 48 | 1 (2.08) | 0 (0) | 47 (97.92) | 41 | 4 (9.76) | 0 (0) | 37 (90.24) |
| Cefepime | 47 | 4 (8.51) | 0 (0) | 43 (91.49) | 48 | 2 (4.17) | 1 (2.08) | 45 (93.75) | 33 | 0 (0) | 4 (12.12) | 29 (87.88) |
| Cefazolin | | | | | | | | | 28 | 20 (71.43) | 0 (0) | 8 (28.57) |
| Doripenem | 47 | 4 (8.51) | 0 (0) | 43 (91.49) | 49 | 3 (6.12) | 1 (2.04) | 45 (91.84) | 32 | 3 (9.38) | 0 (0) | 29 (90.63) |
| Ertapenem | 7 | 0 (0) | 0 (0) | 7 (100) | | | | | 31 | 0 (0) | 0 (0) | 31 (100) |
| Imipenem | 48 | 4 (8.33) | 0 (0) | 44 (91.67) | 50 | 6 (12) | 3 (6) | 41 (82) | 58 | 6 (10.34) | 0 (0) | 52 (89.66) |
| Meropenem | 48 | 4 (8.33) | 0 (0) | 44 (91.67) | 50 | 5 (10) | 0 (0) | 45 (90) | 58 | 5 (8.62) | 2 (3.45) | 51 (87.93) |
| Aztreonam | 0 | | | | 42 | 8 (19.05) | 7 (16.67) | 27 (64.29) | 48 | 9 (18.75) | 3 (6.25) | 36 (75) |
| Tobramycin | 48 | 3 (6.25) | 0 (0) | 45 (93.75) | 52 | 2 (3.85) | 0 (0) | 50 (96.15) | 46 | 12 (26.09) | 2 (4.35) | 32 (69.57) |
| Amikacin | 48 | 5 (10.42) | 1 (2.08) | 42 (87.5) | 52 | 2 (3.85) | 0 (0) | 50 (96.15) | 49 | 3 (6.12) | 0 (0) | 46 (93.88) |
| Gentamicin | 48 | 6 (12.5) | 1 (2.08) | 41 (85.42) | | | | | 49 | 6 (12.24) | 2 (4.08) | 41 (83.67) |
| Tigecycline | | | | | | | | | 30 | 0 | 0 | 30 |

| | | | | | | | | | | | | |
|---|---|---|---|---|---|---|---|---|---|---|---|---|
| | | | | | | | | | | (0) | (0) | (100) |
| Minocycline | 41 | 1 (2.44) | 2 (4.88) | 38 (92.68) | 5 | 1 (20) | 0 (0) | 4 (80) | 60 | 11 (18.33) | 4 (6.67) | 45 (75) |
| Tetracycline | 48 | 6 (12.5) | 0 (0) | 42 (87.5) | | | | | 55 | 24 (43.64) | 2 (3.64) | 29 (52.73) |
| Nitrofurantoin | | | | | | | | | 28 | 12 (42.86) | 3 (10.71) | 13 (46.43) |
| Ciprofloxacin | 39 | 3 (7.69) | 0 (0) | 36 (92.31) | 57 | 5 (8.77) | 1 (1.75) | 51 (89.47) | 62 | 15 (24.19) | 2 (3.23) | 45 (72.58) |
| Levofloxacin | 48 | 4 (8.33) | 1 (2.08) | 43 (89.58) | 57 | 5 (8.77) | 1 (1.75) | 51 (89.47) | 62 | 11 (17.74) | 1 (1.61) | 50 (80.65) |
| Trimethoprim/Sulfamethoxazole | 48 | 4 (8.33) | 0 (0) | 44 (91.67) | 5 | 0 (0) | 0 (0) | 5 (100) | 68 | 14 (20.59) | 0 (0) | 54 (79.41) |

T = number of tested isolates, R = resistant, I = intermediate, S = susceptible.

Table S6: Antibiotic susceptibility profiles of *Staphylococcus aureus*, and coagulase-negative *staphylococci (*CoNS) in the current study.

| Antibiotics | ***Staphylococcus aureus*** | | | | **Coagulase-negative *staphylococci* (CoNS)** | | | |
|---|---|---|---|---|---|---|---|---|
| | T | R (%) | I (%) | S (%) | T | R (%) | I (%) | S (%) |
| Ampicillin | 112 | 101 (90.18) | 0 (0) | 11 (9.82) | 203 | 177 (87.19) | 0 (0) | 26 (12.81) |
| Oxacillin | 111 | 40 (36.04) | 0 (0) | 71 (63.96) | 202 | 135 (66.83) | 0 (0) | 67 (33.17) |
| Penicillin G | 112 | 101(90.18) | 0 (0) | 11 (9.82) | 203 | 179 (88.18) | 0 (0) | 24 (11.82) |
| Gentamicin | 112 | 3 (2.68) | 2 (1.79) | 107 (95.54) | 197 | 20 (10.15) | 8 (4.06) | 169 (85.79) |
| Tigecycline | 111 | 2 (1.8) | 0 (0) | 109 (98.2) | 194 | 0 (0) | 0 (0) | 194 (100) |
| Tetracycline | 112 | 14 (12.5) | 2 (1.79) | 96 (85.71) | 198 | 47 (23.74) | 1 (0.51) | 150 (75.76) |
| Nitrofurantoin | 112 | 0 (0) | 0 (0) | 112 (100) | 198 | 3 (1.52) | 0 (0) | 195 (98.48) |
| Moxifloxacin | 82 | 13 (15.85) | 0 (0) | 69 (84.15) | 128 | 45 (35.16) | 13 (10.16) | 70 (54.69) |
| Levofloxacin | 112 | 18 (16.07) | 1 (0.89) | 93 (83.04) | 202 | 95 (47.03) | 5 (2.48) | 102 (50.5) |
| Ciprofloxacin | 111 | 23 (20.72) | 5 (4.5) | 83 (74.77) | 202 | 100 (49.5) | 10 (4.95) | 92 (45.54) |
| Trimethoprim/Sulfamethoxazole | 112 | 4 (3.57) | 0 (0) | 108 (96.43) | 199 | 66 (33.17) | 0 (0) | 133 (66.83) |
| Vancomycin | 109 | 0 (0) | 0 (0) | 109 (100) | 188 | 1 (0.53) | 0 (0) | 187 (99.47) |
| Chloramphenicol | 112 | 1 (0.89) | 5 (4.46) | 106 (94.64) | 198 | 12 (6.06) | 1 (0.51) | 185 (93.43) |
| Clindamycin | 112 | 13 (11.61) | 0 (0) | 99 (88.39) | 200 | 41 (20.5) | 1 (0.5) | 158 (79) |
| Daptomycin | 107 | 0 (0) | 0 (0) | 107 (100) | 182 | 0 (0) | 0 (0) | 182 (100) |
| Erythromycin | 112 | 50 (44.64) | 1 (0.89) | 61 (54.46) | 204 | 144 (70.59) | 0 (0) | 60 (29.41) |
| Linezolid | 112 | 0 (0) | 0 (0) | 112 (100) | 198 | 0 (0) | 0 (0) | 198 (100) |
| Rifampin | 112 | 2 (1.79) | 3 (2.68) | 107 (95.54) | 198 | 10 (5.05) | 0 (0) | 188 (94.95) |
| Quinupristin/Dalfopristin | 112 | 1 (0.89) | 0 (0) | 111 (99.11) | 198 | 0 (0) | 0 (0) | 198 (100) |

T = number of tested isolates, R = resistant, I = intermediate, S = susceptible.

Table S7: Antibiotic susceptibility profiles of *Enterococcus* species, *Streptococcus* species in the current study.

| **Antibiotics** | ***Enterococcus* species** | | | | ***Streptococcus* species** | | | |
|---|---|---|---|---|---|---|---|---|
| | T | R (%) | I (%) | S (%) | T | R (%) | I (%) | S (%) |
| Ampicillin | 57 | 27 (47.37) | 0 (0) | 30 (52.63) | 5 | 1 (20) | 0 (0) | 4 (80) |
| Penicillin G | 48 | 24 (50) | 0 (0) | 24 (50) | 19 | 2 (10.53) | 0 (0) | 17 (89.47) |
| Tigecycline | 41 | 1 (2.44) | 0 (0) | 40 (97.56) | 22 | 0 (0) | 0 (0) | 22 (100) |
| Tetracycline | 58 | 29 (50) | 0 (0) | 29 (50) | 34 | 12 (35.29) | 0 (0) | 22 (64.71) |
| Nitrofurantoin | 56 | 1 (1.79) | 16 (28.57) | 39 (69.64) | 1 | 0 (0) | 0 (0) | 1 (100) |
| Moxifloxacin | | | | | 16 | 1 (6.25) | 0 (0) | 15 (93.75) |
| Levofloxacin | 58 | 36 (62.07) | 0 (0) | 22 (37.93) | 39 | 3 (7.69) | 0 (0) | 36 (92.31) |
| Ciprofloxacin | 56 | 37 (66.07) | 1 (1.79) | 18 (32.14) | 1 | 0 (0) | 0 (0) | 1 (100) |
| Trimethoprim/Sulfamethoxazole | 2 | 0 (0) | 0 (0) | 2 (100) | 24 | 2 (8.33) | 2 (8.33) | 20 (83.33) |
| Vancomycin | 58 | 0 (0) | 0 (0) | 58 (100) | 35 | 0 (0) | 0 (0) | 35 (100) |
| Chloramphenicol | 58 | 5 (8.62) | 5 (8.62) | 48 (82.76) | 41 | 0 (0) | 0 (0) | 41 (100) |
| Clindamycin | | | | | 38 | 12 (31.58) | 0 (0) | 26 (68.42) |
| Daptomycin | 53 | 2 (3.77) | 5 (9.43) | 46 (86.79) | 21 | 0 (0) | 0 (0) | 21 (100) |
| Erythromycin | 58 | 36 (62.07) | 7 (12.07) | 15 (25.86) | 42 | 18 (42.86) | 2 (4.76) | 22 (52.38) |
| Linezolid | 58 | 9 (15.52) | 1 (1.72) | 48 (82.76) | 38 | 0 (0) | 0 (0) | 38 (100) |
| Rifampin | 56 | 25 (44.64) | 12 (21.43) | 19 (33.93) | | | | |
| Quinupristin/Dalfopristin | 56 | 26 (46.43) | 10 (17.86) | 20 (35.71) | 1 | 0 (0) | 0 (0) | 1 (100) |

T = number of tested isolates, R = resistant, I = intermediate, S = susceptible.

Table S8: Risk factors analysis of MDR versus non-MDR for all bacterial isolates in the current study.

| **Variables** | **MDR (n = 513)** | **MDR (%) 44.18%** | **N-MDR (n = 648)** | **N-MDR (%) 55.18%** | **Odd ratio** | **95% CI** | **p value** |
|---|---|---|---|---|---|---|---|
| **Gender** | | | | | | | |
| Male | 311 | 60.62 | 365 | 56.33 | 1.19 | 0.94 to 1.51 | 0.14 |
| Female | 202 | 39.38 | 283 | 43.67 | 0.84 | 0.66 to 1.06 | 0.14 |
| **Age (Median (IQR)** | 60 | (51 to 67) | 58 | (49 to 67) | -1# | | 0.12* |
| **Occupation** | | | | | | | |
| Jobless/Unemployed/Retired | 152 | 29.63 | 197 | 30.4 | 0.96 | 0.75 to 1.25 | 0.78 |
| Laborer/Manual Worker | 293 | 57.12 | 357 | 55.09 | 1.09 | 0.86 to 1.37 | 0.49 |
| Freelancer/Self-Employed | 34 | 6.63 | 60 | 9.26 | 0.7 | 0.45 to 1.07 | 0.1 |
| Professional and Technical | 34 | 6.63 | 34 | 5.25 | 1.28 | 0.80 to 2.07 | 0.32 |
| **Education background** | | | | | | | |
| No Formal Education | 138 | 26.9 | 180 | 27.78 | 0.96 | 0.74 to 1.24 | 0.74 |
| Basic Education | 170 | 33.14 | 215 | 33.18 | 1 | 0.78 to 1.27 | 0.99 |
| Intermediate Education | 83 | 16.18 | 113 | 17.44 | 0.91 | 0.67 to 1.25 | 0.57 |
| Secondary Education | 93 | 18.13 | 101 | 15.59 | 1.2 | 0.88 to 1.64 | 0.25 |
| Higher Education | 29 | 5.65 | 39 | 6.02 | 0.94 | 0.56 to 1.55 | 0.79 |
| **Type of infection** | | | | | | | |
| Hospital acquired | 428 | 83.43 | 522 | 80.56 | 1.22 | 0.45 to 1.65 | 0.21 |
| Community acquired | 85 | 16.57 | 126 | 19.44 | 0.82 | 0.61 to 1.12 | 0.21 |
| **Cancer treatment** | | | | | | | |
| Chemotherapy | 289 | 56.34 | 380 | 58.64 | 0.91 | 0.72 to 1.15 | 0.43 |
| Radiotherapy | 55 | 10.72 | 72 | 11.11 | 0.96 | 0.66 to 1.40 | 0.83 |
| Targeted therapy | 93 | 18.13 | 137 | 21.14 | 0.83 | 0.61 to 1.11 | 0.2 |
| Immunotherapy | 45 | 8.77 | 57 | 8.8 | 1 | 0.66 to 1.50 | 0.99 |
| **Procedures and Interventions** | | | | | | | |
| CVC | 72 | 14.04 | 86 | 13.27 | 1.07 | 0.77 to 1.50 | 0.71 |
| Urinary catheter | 146 | 28.46 | 117 | 18.06 | 1.81 | 1.37 to 2.37 | **<0.001** |
| PICC | 93 | 18.13 | 103 | 15.9 | 1.17 | 0.86 to 1.60 | 0.31 |
| Safety IV catheter | 289 | 56.34 | 380 | 58.64 | 0.91 | 0.72 to 1.15 | 0.43 |
| Ventilator | 30 | 5.85 | 19 | 2.93 | 2.06 | 1.17 to 3.71 | **0.01** |
| Blood transfusion | 133 | 25.93 | 141 | 21.76 | 1.26 | 0.96 to 1.65 | 0.1 |

| | | | | | | | |
|---|---|---|---|---|---|---|---|
| Biopsies | 51 | 9.94 | 67 | 10.34 | 0.96 | 0.66 to 1.40 | 0.82 |
| Intra-abdominal catheter | 87 | 16.96 | 91 | 14.04 | 1.25 | 0.91 to 1.72 | 0.17 |
| Parenteral nutrition | 161 | 31.38 | 178 | 27.47 | 1.21 | 0.94 to 1.55 | 0.15 |
| Nasogastric feeding | 43 | 8.38 | 31 | 4.78 | 1.82 | 1.14 to 2.91 | **0.01** |
| Surgery | 73 | 14.23 | 64 | 9.88 | 1.51 | 1.07 to 2.16 | **0.02** |
| **Infection and infectious diseases** | | | | | | | |
| Sepsis | 249 | 48.54 | 321 | 49.54 | 0.96 | 0.76 to 1.21 | 0.74 |
| Septic shock | 39 | 7.6 | 43 | 6.64 | 1.16 | 0.73 to 1.82 | 0.52 |
| Hepatitis B | 71 | 13.84 | 83 | 12.81 | 1.09 | 0.78 to 1.55 | 0.61 |
| Pneumonia | 82 | 15.98 | 103 | 15.9 | 1.01 | 0.73 to 1.37 | 0.97 |
| Urinary tract infection | 48 | 9.36 | 25 | 3.86 | 2.57 | 1.58 to 4.22 | **<0.001** |
| Biliary tract infection | 44 | 8.58 | 49 | 7.56 | 1.15 | 0.74 to 1.75 | 0.53 |
| **Conditions and complications** | | | | | | | |
| Anemia | 151 | 29.43 | 174 | 26.85 | 1.14 | 0.88 to 1.47 | 0.33 |
| Hypertension | 75 | 14.62 | 102 | 15.74 | 0.92 | 0.67 to 1.27 | 0.6 |
| Diabetes | 45 | 8.77 | 59 | 9.1 | 0.96 | 0.64 to 1.44 | 0.84 |
| Thrombosis | 15 | 2.92 | 25 | 3.86 | 0.75 | 0.40 to 1.41 | 0.39 |
| Myelosuppression | 140 | 27.29 | 166 | 25.62 | 1.09 | 0.84 to 1.41 | 0.52 |
| Hypoproteinemia | 214 | 41.72 | 215 | 33.18 | 1.44 | 1.14 to 1.83 | 0.002 |
| Nausea and Vomiting | 20 | 3.9 | 18 | 2.78 | 1.42 | 0.74 to 2.67 | 0.29 |
| Radiation dermatitis | 9 | 1.75 | 10 | 1.54 | 1.14 | 0.47 to 2.82 | 0.78 |
| Respiratory failure | 25 | 4.87 | 23 | 3.55 | 1.39 | 0.78 to 2.51 | 0.26 |
| Ascites | 66 | 12.87 | 71 | 10.96 | 1.2 | 0.84 to 1.70 | 0.32 |
| Leukopenia | 54 | 10.53 | 71 | 10.96 | 0.96 | 0.66 to 1.40 | 0.81 |
| Cerebral infarction | 13 | 2.53 | 25 | 3.86 | 0.65 | 0.32 to 1.27 | 0.21 |
| Electrolyte disorders | 107 | 20.86 | 115 | 17.75 | 1.22 | 0.91 to 1.63 | 0.18 |
| Neutropenia | 62 | 12.09 | 95 | 14.66 | 0.8 | 0.56 to 1.13 | 0.2 |
| Neutrophilia | 206 | 40.16 | 269 | 41.51 | 0.95 | 0.75 to 1.20 | 0.64 |
| **Hospital stays (in days)** | 21 | (13 to 32) | 19 | (13 to 30) | -2# | | 0.12* |
| **Mortality (30 days)** | 31 | 6.04 | 27 | 4.17 | 1.48 | 0.89 to 2.52 | 0.15 |

Footnotes: *Mann-Whitney U test; #Difference: Hodges-Lehmann; CVC, Central Venous Catheter; PICC, Peripherally Inserted Central Catheter
